# The Role of Hydrogen Sulfide in Plant Roots during Development and in Response to Abiotic Stress

**DOI:** 10.3390/ijms23031024

**Published:** 2022-01-18

**Authors:** Hua Li, Hongyu Chen, Lulu Chen, Chenyang Wang

**Affiliations:** 1 College of Life Science, Henan Agricultural University, Zhengzhou 450002, China; CHY19970927@163.com (H.C.); Nancy061798@163.com (L.C.); 2 State Key Laboratory of Crop Biology, Shandong Agricultural University, Taian 271018, China; 3 College of Agronomy, Henan Agricultural University, Zhengzhou 450002, China; 4 State Key Laboratory of Wheat and Maize Crop Science, Henan Agricultural University, Zhengzhou 450002, China

**Keywords:** hydrogen sulfide, root growth, nitric oxide, auxin, heavy metal, salt

## Abstract

Hydrogen sulfide (H_2_S) is regarded as a “New Warrior” for managing plant stress. It also plays an important role in plant growth and development. The regulation of root system architecture (RSA) by H_2_S has been widely recognized. Plants are dependent on the RSA to meet their water and nutritional requirements. They are also partially dependent on the RSA for adapting to environment change. Therefore, a good understanding of how H_2_S affects the RSA could lead to improvements in both crop function and resistance to environmental change. In this review, we summarized the regulating effects of H_2_S on the RSA in terms of primary root growth, lateral and adventitious root formation, root hair development, and the formation of nodules. We also discussed the genes involved in the regulation of the RSA by H_2_S, and the relationships with other signal pathways. In addition, we discussed how H_2_S regulates root growth in response to abiotic stress. This review could provide a comprehensive understanding of the role of H_2_S in roots during development and under abiotic stress.

## 1. Introduction

The root system is an important vegetative organ of plants. In terrestrial environments, the root system provides structural support, uptakes water and nutrition from soil, and is where some amino acids, endogenous hormones, and other substances are synthesized. The growth and development of the root system largely determines the water and nutrient absorption efficiency of plants. With the improvement of genomics, genetics, molecular biology, and other research methods, as well as the generation of a mutant library related to root development, more and more functional genes and regulatory genes that affect root growth have been identified [1,2]. In addition to being regulated by internal genes, the physical environment can also have a regulatory effect on the growth and development of plant roots, such as the soil temperature, moisture, nutrients, and pH [3,4,5,6,7]. Plant signal molecules act as a bridge between the physical environment and root-growth-related genes, and hence determine how plants respond to environmental stress and changes. The plant signal molecules regulate the expression of root-growth-related genes through the transmission and transduction of environmental signals, which can lead to changes in the root system architecture (RSA) (including primary growth, the formation of lateral or adventitious roots, and the distribution and length of root hairs). These signal molecules include plant hormones, nitric oxide (NO), carbon monoxide (CO), reactive oxygen species (ROS), and hydrogen sulfide (H_2_S). The importance of plant hormones, NO, and ROS to root growth and development has been reviewed in many articles [8,9,10,11,12,13,14]. Hydrogen sulfide plays an important role in plant growth and development, and responds to various environmental stresses. In the past ten years, more than 2000 literatures have reported and discussed the impact of H_2_S on plant physiology. The importance of H_2_S for regulating plant responses to abiotic stresses (such as drought, salt, heat, and heavy metals) [15,16,17] and the effects on stomatal movement, seed germination, leaf senescence, and fruit ripening [18,19] have been extensively studied and discussed. In this review, we focused on the role of H_2_S in root growth and development. The effects of H_2_S on primary root growth, lateral and adventitious root formation, root hair development, and nodules were summarized here. As H_2_S can alleviate the negative impacts of abiotic stress on plant root growth, we also reviewed how H_2_S regulates root growth in response to abiotic stress. 

## 2. The Role of Hydrogen Sulfide during Root Development

**Figure 1:**
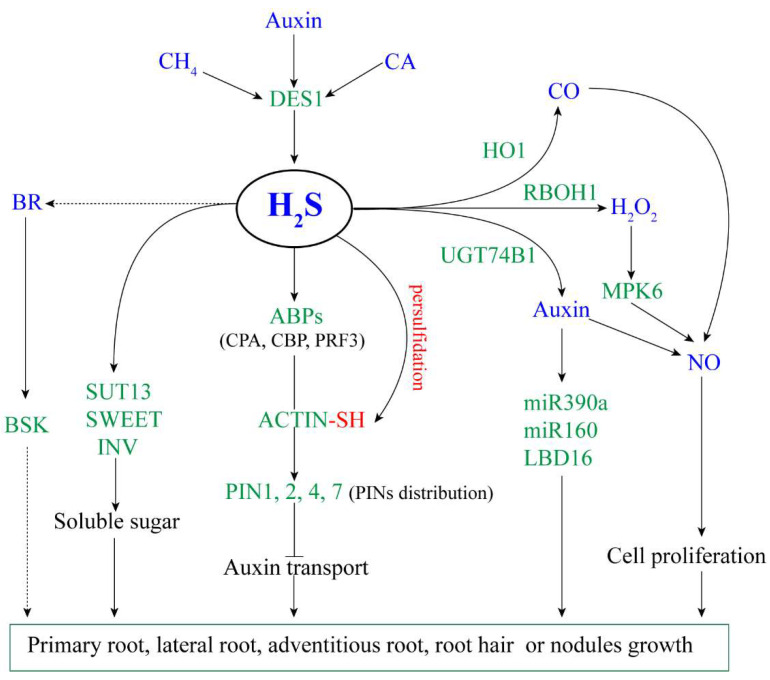
The proposed model of H_2_S regulating plant root growth. Arrow and bar ends indicate activation and inhibitory effects, respectively. Green fonts represent genes or proteins, blue fonts represent signal molecules. H_2_S: hydrogen sulfide; CH_4_: methane; CA: cinnamaldehyde; BR: brassinosteroid; CO: carbon monoxide; NO: nitric oxide; DES1: L-cysteine desulfhydrase 1; HO1: haem oxygenase-1; RBOH1: respiratory burst oxidase 1; UGT74B1: UDP-glycosyltransferase 74B1; MPK6: mitogen-activated protein kinase 6; ABPs: actin-binding proteins; PINs: pin-formed family; SUT13: sugar transport protein 13; SWEET: bidirectional sugar transporter; INV: invertase; BSK: BR-signaling kinase; LBD16: LOB domain-containing protein 16.

The literature on the regulation of H_2_S on root growth and development were shown in Table 1. According to these studies, we summarized a model for the regulatory mechanism of H_2_S on root growth and development (Figure 1).

### 2.1. Hydrogen Sulfide Regulates the Formation and Growth of Lateral and Adventitious Roots 

Hydrogen sulfide has a concentration-dependent effect on the regulation of root growth. In tomato, 0.01–1.0 mM sodium hydrosulfide (NaHS) (a H_2_S donor) can significantly promote the initiation and length of lateral roots (LRs) and can increase the number and density of LRs. However, a high concentration of NaHS (10 mM) inhibits the LRs’ growth [23]. A similar phenomenon was found in the mangrove plant *Kandelia obovata*, where a concentration of 0.01–1.0 mM NaHS led to a notable increase in the length and total surface area of LRs [27]. H_2_S was essential for the formation of pepper LRs, where a concentration of 0.5–8.0 mM NaHS significantly increased the number of LRs. In contrast, different concentrations of the H_2_S scavenger hypotaurine (HT) markedly inhibited the formation of LRs [24]. In peach, H_2_S had a notable effect on the formation of LRs, with a concentration of 0.2 mM NaHS leading to a significant increase in the number of LRs [33]. Our previous research also found that H_2_S promoted the growth and development of lateral roots in wheat, with a concentration of 0.4 mM NaHS resulting in an increase in the number, density, and length of LRs [35]. However, in *Arabidopsis*, the effects of H_2_S on lateral roots were slightly different. H_2_S can promote the occurrence of LRs, but inhibits the LRs’ length [32], which may be related to the concentration of NaHS used in the treatment.

The effect of H_2_S on the formation and growth of adventitious roots is the same as that for lateral roots. The application of the appropriate concentration of H_2_S promoted the number and length of adventitious roots in sweet potato [20]. In addition, the same result was obtained for excised willow, soybeans [20], and cucumber [21,22].

### 2.2. Hydrogen Sulfide Inhibits the Growth of Primary Roots and Root Hair 

Several studies have shown that H_2_S has a toxic effect on the growth of primary roots. The concentration of exogenous NaHS used in treatments was negatively correlated with the rate of growth (length) of primary roots [28,29,32]. This inhibitory effect of H_2_S on primary roots may be related to the reduction in the meristem cell division potential, as the length of the root meristematic zones were reduced when treated with NaHS [28]. Although H_2_S inhibited the length of root meristematic zones, Li et al. [29] found that the length from the root apex to a root hair for the seedlings was promoted by H_2_S. This may be due to the inhibitory effect of H_2_S on root hairs. H_2_S is known to inhibit the initiation of root hair; that is, the starting site of the root hair zones may be further away from the root apex [29], resulting in a longer distance from the root hair to root apex, even when H_2_S inhibits the meristem zones. In addition, H_2_S not only inhibited the initiation of root hair growth, but also significantly decreased the root hair length and density [29].

### 2.3. Hydrogen Sulfide Promotes the Formation of Root Nodules and Nitrogen Fixation

Root nodules are a special organ formed by symbiosis between leguminous plants (Fabaceae) and rhizobia. The formation and growth of the nodules are strictly controlled by plant hormones [36]. As a recognized signal molecule that interacts with plant hormones to regulate plant growth and development, H_2_S is known to influence the growth of root nodules [30,31]. Endogenous H_2_S production in both young soybean nodules (14 days post-inoculation [DPI] with the *Sinorhizobium fredii Q8* strain) and mature nodules (28 DPI) can be detected by fluorescent probes SF7-A, whereas no significant fluorescence was observed in the nascent soybean nodules (7 DPI). This suggested that H_2_S may mediate the growth of root nodules [31]. Indeed, the application of NaHS significantly increased the number of soybean nodules and enhanced nitrogenase (Nase) activity after 7 DPI and 24 DPI, respectively. In addition, H_2_S was found to affect rhizobial infection, where a greater abundance of developing infection threads and cortex infection threads was found in NaHS-treated soybean roots than those in untreated controls at 5 DPI and 7 DPI, respectively [30]. On the contrary, an endogenous H_2_S production deficit rhizobia mutant ΔCSE (cystathionine γ-lyase) symbiosis with soybean roots significantly reduced the nitrogenase activity and H_2_S content in nodule cells. Moreover, higher contents of H_2_O_2_ (hydrogen peroxide), MDA (malondialdehyde), and protein carbonyl were observed in ΔCSE root nodules; that is, the H_2_S-induced nitrogen-fixation ability of root nodules may be related to its regulation of the antioxidant system that protects nodule cells from oxidative damage [31]. These studies suggested that H_2_S might have a positive effect on the soybean–rhizobium symbiosis system and may enhance nitrogen fixation. 

### 2.4. Hydrogen Sulfide Interacts with Other Signaling Molecules to Regulate Root Development

#### 2.4.1. Auxin

The inhibition of primary root growth by H_2_S, and the promotion of lateral and adventitious root formation was consistent with the known effects of auxin on root development. It is not difficult to associate H_2_S and auxin signaling to RSA. The change in the endogenous IAA (indole acetic acid) content was similar to that reported for H_2_S, but with different time-courses in sweet potato explants. The increase in the H_2_S content during the formation of sweet potato adventitious roots preceded changes to the IAA content [20]. The research of Wu et al. (2021) [33] on peach roots also obtained similar results: NaHS induced a significant increase in the endogenous H_2_S content in roots at 1 DAT (days after treatment), while it increased the concentration of endogenous auxin in roots by 44.50% at 5 DAT. Moreover, it was found that treatment with NaHS significantly increased the production of IAA, and that N-1-naphthylphthalamic acid (an IAA transport inhibitor, NPA) weakened the effect of H_2_S on the number of adventitious roots in sweet potato, soybean, and willow [20]. These results showed that IAA may be located downstream of H_2_S in order to mediate root development. However, the results in tomato indicated H_2_S might partially act as a downstream component of the auxin signaling to trigger lateral root formation [23]. The depletion of auxin down-regulated the transcription of *SlDES1* (L-cysteine desulfhydrase 1, a H_2_S synthesis gene), DES activity, and endogenous H_2_S contents in tomato roots, and the inhibitory effect of NPA on lateral root formation was offset by NaHS, whereas the inhibition of lateral root formation by HT was not reversed by naphthalene acetic acid (NAA) [23]. In addition, H_2_S not only induced auxin synthesis, but also affected the auxin response and transport. After the application of NaHS, the expression of the indicator of the auxin response DR5::GUS (synthetic auxin-responsive promoter::β-glucuronidase) was attenuated in the quiescent center (QC), columella initial cells, and mature columella cells of the root apex, and was concentrated to the QC [32]. The movement of auxin in the root acropetal and basipetal was reduced by an increase in the NaHS concentration, which implied that an increase in H_2_S levels reduces the IAA transport capacity. Further research showed that the inhibition of IAA transport by H_2_S was related to the polar subcellular localization of PIN proteins (PIN1, PIN2, PIN4, and PIN7) [32].

#### 2.4.2. Reactive Oxygen Species

High concentrations of ROS (reactive oxygen species) often cause oxidative damage to plants, but low concentrations of ROS are necessary for signaling to maintain plant growth and development. The ROS-related regulation of root development has been reported for *Arabidopsis* [37], tomato [38], maize [39,40], and sweet potato [41]. The relationship between ROS and H_2_S for the regulation of root growth was also discussed in several studies [25,28,34]. These studies found that ROS signaling might be downstream of H_2_S to mediate RSA. For example, H_2_S could induce the expression of *RBOH1* (respiratory burst oxidase 1) in tomato roots and could enhance the accumulation of H_2_O_2_, thereby promoting lateral root formation. These H_2_S-related effects on lateral roots were destroyed by DMTU (dimethylthiourea, a H_2_O_2_ scavenger) and DPI (diphenylene idonium, an inhibitor of NADPH oxidase) [25]. The inhibitory effect of H_2_S on primary root growth depended on the ROS pathway, as the relative root growth in *rbohF* and *rbohD/F* was higher than that in WT for the NaHS treatment, which meant that respiratory burst oxidase homolog mutants (*rboh*) were less sensitive to treatment with NaHS [28]. The promoting effect of H_2_S on strawberry roots during plug transplant production could also be attributed (in part) to the elevated H_2_O_2_ [34].

#### 2.4.3. Nitric Oxide and Carbon Monoxide

Nitric oxide (NO), carbon monoxide (CO), and H_2_S are the three gas signal molecules in organisms. NO and CO also participate in root growth and development [42,43,44,45,46,47]. Therefore, the relationship between H_2_S and NO or CO has attracted attention in the regulation of RSA. The H_2_S-mediated adventitious root formation was alleviated by 2-(4-carboxyphenyl)-4,4,5,5-tetramethylimidazoline-1-oxyl-3-oxide (cPTIO, an NO scavenger) in sweet potato, willow, and soybean [20]. The toxic effect of H_2_S on the primary root of *Arabidopsis* was reduced in NO synthase mutants (*nia1/2* and *noa1*), or when treated with cPTIO and NG-nitro-L-Arg-methyl ester (L-NAME, NO synthesis inhibitor) [28]. These results indicated that H_2_S acts upstream of NO signal transduction pathways when regulating adventitious root formation and primary root growth. From the results reported by Lin et al. (2012) [21], it is known that haem oxygenase-1/carbon monoxide (HO-1/CO) also acts as a downstream signal system during H_2_S-induced adventitious root formation. NaHS up-regulated *HO1* gene expression and promoted HO1 protein accumulation, thereby increasing the number of cucumber adventitious roots. These phenomena were inhibited by ZnPPIX (zinc protoporphyrin IX, an inhibitor of HO-1), whereas the removal of H_2_S by HT did not affect the CO-induced adventitious rooting.

#### 2.4.4. Brassinosteroid, Methane, and Cinnamaldehyde

Brassinosteroid (BR) contributes to the maintenance of root meristems, root cell elongation, lateral root development, root hair formation, and rhizosphere symbiosis [48,49,50,51]. At present, there is no direct evidence that H_2_S interacts with BR to regulate root development, but a recent proteomic analysis in *Kandelia obovata* has shown that H_2_S induced the accumulation of the BR-positive regulator protein BSK [27]. An RNA-seq analysis also showed that differentially expressed genes (DEGs) in peach roots, regulated by H_2_S, were significantly enriched in the “Brassinosteroid biosynthesis” pathway [33]. These results implied that H_2_S-mediated RSA might depend on the BR signal pathway.

Methane (CH_4_) plays an important role in the response to abiotic stress (such as heavy metal, salinity, and osmotic stress) [52]. In recent years, the role of CH_4_ in the formation of lateral and adventitious roots has been elucidated [22,26,53,54,55,56]. Both NO and CO signaling pathways were involved in CH_4_-induced adventitious root formation in cucumber [53,54]. Hydrogen peroxide (H_2_O_2_) signaling is also known to mediate the effects of CH_4_ on tomato lateral root formation [56]. As expected, H_2_S was confirmed to be located downstream of CH_4_ in order to regulate adventitious and lateral root formation in both cucumber and tomato. Methane induced the DES enzyme activity and promoted the production of endogenous H_2_S. These methane-related effects on the adventitious roots of cucumber were blocked by HT [22]. The same results were reported for the relationship between CH_4_ and H_2_S on the formation of lateral roots in tomato [26].

Cinnamaldehyde (CA) is a natural plant essential oil with antibacterial properties. It is widely used as a food additive and in medicines [57]. Recently, CA has also been used as a biological agent for plant disease resistance. For example, CA showed significant antibacterial activity against *Pseudomonas syringae pv. actinidiae*, which causes bacterial canker disease in kiwifruit [58]. Cinnamaldehyde reduced the number of *Meloidogyne incognita* galls and eggs on the roots of soybean plants to approximately 14% and 7%, respectively [59]. In addition, CA was found to play an important role in root development, as it markedly induced the formation of lateral roots in pepper, but without any inhibitory effect on primary root growth. Further study showed that H_2_S participated in this regulation process. Cinnamaldehyde increased the DES activity and promoted endogenous H_2_S production, thereby increasing the number of lateral roots. However, treatment with HT counteracted the effect of CA on endogenous H_2_S and lateral roots [24]. 

### 2.5. The Genes Involved in Hydrogen Sulfide-Mediated Root Development

Root system architecture is continuously adjusted in response to changes in various endogenous and exogenous factors (such as plant hormones, light, nutrition, and water). The regulation of these factors on root development involves many genes, including genes related to auxin synthesis, transport, and response, and genes related to cytokinin, abscisic acid (ABA), nitrate sensing and transport, and photoreceptors. The roles of these genes in plant growth and development were reviewed by Satbhai et al. (2015) [60]. In addition, many miRNAs are also involved in root development and architecture [2]. It is therefore important for researchers to have a clear understanding of which genes are involved in H_2_S signaling, and hence the regulation of root development. We have carried out a detailed discussion and summary of gene regulation below.

#### 2.5.1. Genes Associated with the Auxin Signaling Pathway

The RNA-seq results for peach roots showed that 963 and 1113 DEGs were detected after H_2_S treatments for 1 day and 5 days, respectively [33]. These DEGs were significantly enriched in the “Glutathione metabolism”, “Plant-pathogen interaction”, “Plant hormone signal transduction”, “Brassinosteroid biosynthesis”, and “Cyanoamino acid metabolism” pathways. In particular, the pathway for “Plant hormone signal transduction” was significantly enriched when treated with H_2_S for 1 day and 5 days. A significant proportion (73.68%) of the genes associated with this pathway were related to auxin. More specifically, there were 2, 7, and 17 genes involved in auxin biosynthesis, transport, and signal transduction, respectively. These auxin-related genes included *UGT74B1*, *TAA1*, *PINs*, *ABCBs*, *ARFs*, *Aux/IAAs*, *GH3*, and *SAUR*. The auxin-synthesis-related gene *UGT74B1* was up-regulated 1.95-fold when subjected to the H_2_S treatment. This might explain the H_2_S-induced increase in the root auxin content [20,33]. *PINs* exhibited different expression patterns over time under the NaHS treatment. After treatment with NaHS, *PIN1* was up-regulated during 3 to 6 h and recovered to the control levels by 6 h, and the expression of *PIN2* and *PIN7* increased during 3 to 6 h, whereas it decreased in 12 or 24 h. On the contrary, the expression of *PIN4* decreased after being treated with NaHS for 3 to 12 h, but recovered by 24 h. Although H_2_S had different effects on the expression of the *PIN* genes, its effect on the subcellular distribution of the PIN proteins was consistent. H_2_S disrupted the polar distribution of the PIN proteins (PIN1, PIN2, PIN4, and PIN7) on the plasma membrane in the root epidermal cells, and a large amount of PIN::GFP signals were found to dissociate from the plasma membrane upon cytoplasmic entry. Therefore, H_2_S inhibited auxin transport through its effect on the polarity distribution of PIN proteins, thus promoting the initiation of lateral roots [32]. It has been noted that the location of PIN proteins on the membrane was affected by F-actin [61,62], while H_2_S significantly reduced the occupancy rate of F-actin bundles in each cell. This led to the disappearance of thick actin cables [32]. This implied that the influence of H_2_S on the distribution of PIN proteins depended on the actin cytoskeleton, which is directly controlled by different ABPs (actin-binding proteins) [63]. Therefore, the expression of *ABPs* (*CPA*, *CBP*, and *PRF3*) was found to be up-regulated by H_2_S, whereas the effects of H_2_S on the percentage occupancy of the F-actin bundles was partially removed in the *cpa*, *cbp*, and *prf3* mutants [32]. In addition, some auxin signal transduction genes were found to be regulated by H_2_S during root development. *CsAux22D-like* and *CsAux22B-like* were up-regulated by H_2_S during the formation of cucumber adventitious roots [22]. Hydrogen sulfide induced *miR390a* and *miR160*, and thus inhibited the expression of their target genes *ARF4* and *ARF16* in both tomato and *Arabidopsis* roots [25,26]. *AtGATA23* and *AtLBD16* were down-regulated in the *Atdes1* mutant compared to WT, whereas *AtGH3.1* and *AtIAA28* were up-regulated in the *Atdes1* mutant [26].

#### 2.5.2. Genes Associated with Cell Proliferation

Cell proliferation is the basis for root growth and development, so the expression of cell-proliferation-related genes is very important during root growth. In the tomato root, H_2_S up-regulated *SlCDKA;1*, *SlCYCA2;1*, and *AtCYCA2;3*, but down-regulated *SlKRP2* and *AtKRP2* [25,26]. These genes are involved in the cell cycle. *DNAJ-1*, a gene phase that specifically regulates the G2/M cell cycle, was significantly induced by H_2_S in cucumber roots [21,22]. In addition, the expression of *CsCDC6* (a cell-division-related gene) also increased in response to the NaHS treatment [22]. Interestingly, these cell proliferation-related genes also responded to auxin, CO, and CH_4_, which are closely related to the H_2_S signaling pathway. From the results of the RNA-seq work on peach roots, researchers identified that three cyclin genes and thirteen cell wall formation and remodeling-related genes were regulated by H_2_S [33]. All three cyclin genes (*LOC109950471*, *LOC18790988*, and *LOC18784990*) were up-regulated by H_2_S. In contrast, the cell wall formation and remodeling-related genes showed different patterns of expression in response to the H_2_S treatment [33].

#### 2.5.3. Transcription Factors and Protein Kinases

Both transcription factors (TFs) and protein kinases are regulatory genes that mediate plant growth and development. Wu et al. (2021) [33] found that 36 transcription factors in peach roots were regulated by H_2_S, including LBD, MYB, and the AP2/ERF family. The overexpression of the peach *PpLBD16*, which was induced by H_2_S, significantly increased the number of lateral roots in *Arabidopsis*, whereas the *Arabidopsis* mutant *ldb16* and *ldb18* showed a decrease in the number of lateral roots [64]. These results strengthened our understanding of LBD-mediated lateral root growth. Interestingly, LBDs (such as *AtLBD16*, *AtLBD18*, and *AtLBD29*) have been shown to be directly regulated by *ARFs* when regulating the formation of lateral roots [65,66], which implies that H_2_S may interact with the auxin signaling pathway to regulate the growth of lateral roots, partly dependent on *LBD* genes. In *Kandelia obovata* roots, other TFs were also found to respond to H_2_S, such as trihelix transcription factor GT-3b (*GT-3B*), the zinc finger CCCH domain-containing protein 14 (*ZC3H14*), and the MADS-box transcription factor [27].

Previous studies have shown that several protein kinases respond to H_2_S during root development. The calmodulin kinases *CsCDPK1* and *CsCDPK5* were up-regulated by H_2_S in cucumber roots [21]. MPK6 was involved in H_2_S-inhibited primary root growth. When subjected to the NaHS treatment, the root length of the mutant *mpk6* was significantly longer than that for WT. Moreover, MPK6 was shown to function downstream of H_2_S-induced ROS and upstream of NO [35]. In addition, in peach roots, the DEGs in the H_2_S treatment for five days were significantly enriched in the mitogen-activated protein kinase (MAPK) signaling pathway, relative to the control group [33]. These results suggested that *CDPK* and *MAPK* may play an important role in H_2_S-regulated root development.

#### 2.5.4. Genes Associated with Carbohydrate Metabolism

Hu et al. (2020) [34] reported that H_2_S induced the accumulation of soluble sugar in strawberry roots during plug production. Subsequently, the transcriptome and proteome data showed that the H_2_S-regulated genes in roots were significantly enriched in “Starch and sucrose metabolism” [27,33]. These data indicated that soluble sugar was either directly or indirectly involved with H_2_S-regulated root development. The sucrose transport protein SUT13, bidirectional sugar transporter SWEET, and invertase (INV) were found to be up-regulated by H_2_S in *Kandelia obovata* roots, which led researchers to speculate that H_2_S may facilitate sucrose transport and promote the hydrolysis of sucrose to provide metabolites and energy for root growth.

## 3. The Role of Hydrogen Sulfide in Roots Exposed to Abiotic Stress

**Figure 2:**
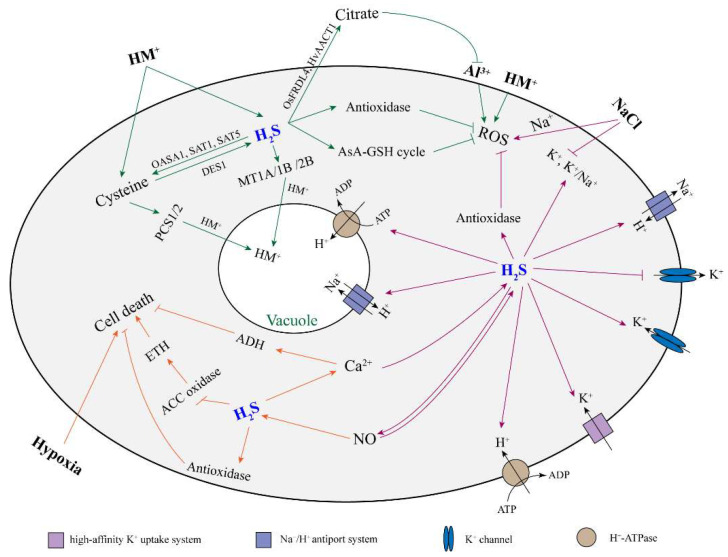
The proposed model of H_2_S alleviating plant root cell damage under abiotic stress. Arrow and bar ends indicate activation and inhibitory effects, respectively. The green, orange, and purple arrows refer to the response of H_2_S in plant roots to heavy metals, hypoxia, and salt stress, respectively. H_2_S: hydrogen sulfide; HM: heavy metal; ROS: reactive oxygen species; NO: nitric oxide; AsA-GSH cycle: ascorbate–glutathione cycle; ETH: ethylene; ADH: alcohol dehydrogenase; DES1: L-cysteine desulfhydrase 1; OASA1: o-acetylserine lyase isoform A1; SAT1: serine acetyltransferase 1; SAT5: serine acetyltransferase 5; PCS1/5: phytochelatin synthase 1/5; MT1A/1B/2B: metallothionein 1A/1B/2B; FRDL4: citrate efflux transporter; AACT1: citrate transporter.

Abiotic stress often stimulates oxidative damage by generating ROS, which leads to the inhibition of plant growth and even death. Plant root growth is sensitive to abiotic stress factors in the soil, such as heavy metals (HMs), aluminum, salinity, and hypoxia. It has been reported that H_2_S could alleviate the inhibitory effect of abiotic stress on root growth in many plants (Table 2). Here, we discussed and reviewed the role and mechanism of H_2_S on root growth when exposed to an abiotic stress (Figure 2).

### 3.1. Heavy Metals

Cadmium (Cd) is regarded as the most toxic of the heavy metals (HMs) for plants. The function of H_2_S in plants subject to Cd-related stress has been extensively studied. Hydrogen sulfide could alleviate the Cd-induced inhibition of root growth in *Arabidopsis* [69], *Medicago sativa* [67,77], *Brassica rapa* [68,70,76], *Setaria italica* [71], *Cucumis sativus* [72,75], *Solanum lycopersicum* [74], and *Hordeum vulgare* [73]. Furthermore, the suppression of plant root growth caused by other heavy metals, such as chromium (Cr), lead (Pb), mercury (Hg), and nickel (Ni), could also be relieved when exposed to H_2_S [73,78,79,82,83,84,92,93]. 

When plants are exposed to HM-related stress, they first reduce the absorption of the HMs, or translocate the HMs to vacuoles to reduce oxidative damage to cells. As expected, the alleviation of the HM-related stress by H_2_S is partly dependent on these pathways. The Cd content in NaHS-pretreated root tissues was 33–37% lower than that for untreated Cd-stressed plant samples [67]. Pretreatment with NaHS markedly reduced the Pb content in maize roots [83]. The Ni content declined in the NaHS+Ni treatment of zucchini roots in comparison to the alone treatment of Ni-stressed [84]. Hydrogen sulfide also had a significant inhibitory effect on the absorption of Cr in cauliflower roots, stems, leaves, and flowers [79].

One of the most significant effects of HMs on plants is the production of large amounts of ROS, which, in turn, leads to oxidative damage to cells. The role of H_2_S in oxidative stress has been a major research focus for many years. Not surprisingly, the antioxidant function of H_2_S plays an important role in the response of plants to exposure to HM stress. When subjected to Cd-stress, treatment with NaHS reduced the accumulation of ROS and lipid peroxidation in *Brassica rapa* and barley [68,73]. The antioxidant function of H_2_S also had an effective response to Cr, Pb, and Hg-induced stress [78,79,82,83,93]. Generally, H_2_S reduced oxidative damage to cells, mainly by inducing antioxidant-related enzyme activity. The NaHS pretreatment significantly increased the activity of SOD (superoxide dismutase), APX (ascorbate peroxidase), CAT (catalase), and POD (peroxidase) in Cr-stressed cauliflower [79]. In addition, the positive effect of H_2_S on anti-oxidation was attributed to glutathione (GSH) homeostasis. The treatment with Cd significantly decreased the content of GSH and homoglutathione (hGSH) and increased the content of GSSG (oxidized GSH) and hGSSGh (oxidized hGSH), whereas the decreased ratio of hGSH/hGSSGh and GSH/GSSG in alfalfa seedlings was obviously inhibited by H_2_S [77]. In the presence of HT, the activities of the AsA-GSH-cycle-related enzymes (ascorbate peroxidase APX, monodehydroascorbate reductase MDHAR, dehydroascorbate reductase DHAR, and glutathione reductase GR) were reduced compared to the activities in the untreated Cd-stressed group, and thus the ratio of AsA/DHA (ascorbate/ dehydroascorbate) and GSH/GSSG declined [74].

In plants, H_2_S is mainly produced from cysteine desulfhydrylase (CDes) catalyzing the degradation of cysteine (Cys). Like H_2_S, when exposed to HM stress, the endogenous Cys content increased in *Arabidopsis* roots. The addition of exogenous Cys can significantly alleviate the inhibitory effect of HMs on primary growth. Cys synthesis could be induced by H_2_S by up-regulating the expression of the Cys-generation-related genes *OASTLa*, *SAT1,* and *SAT5*. Subsequently, Cys promoted GSH accumulation and induced the expression of phytochelatin (PC) genes (*PCS1* and *PCS2*) counteracting Cd^2+^ and Cr^6+^ toxicity, and H_2_S could up-regulate the metallothionein gene (*MT1A*, *MT1B*, *MT2B*) to alleviate the Cd^2+^ and Cr^6+^ toxicity [69,78]. These results indicated that the H_2_S-Cys cycle system played a key role in plant responses to HM-related stress.

Hydrogen sulfide interacted with other signaling molecules to regulate heavy-metal-induced oxidative damage to cells. For example, the accumulation of NO was enhanced by NaHS treatment during exposure to Cd-induced stress. Hydrogen sulfide reduced Cd-induced oxidative damage in alfalfa roots, which was reversed by cPTIO [67]. MeJA enhanced Cd tolerance and alleviated growth inhibition in foxtail millet, whereas these effects were weakened by HT [71]. Similarly, the effects of CH_4_ on redox imbalance and cell death in alfalfa roots subjected to Cd stress was dependent on the induction of H_2_S metabolism. Treatment with either HT or PAG (propargylglycine, a H_2_S biosynthesis inhibitor) reduced the alleviating effects of CH_4_ on Cd-stressed plants [77].

### 3.2. Aluminum

Like heavy metal stress, excessive aluminum (Al) can also cause a large amount of ROS production in cells, resulting in oxidative damage and even cell death. Several studies have reported that H_2_S can weaken the inhibitory effect of aluminum toxicity on plant root growth by inhibiting Al^3+^ absorption and enhancing the antioxidant system. For example, both in barley and rice, the Al content in the leaves and roots of Al-stressed plants treated with NaHS was much lower than for untreated plants [80,81,94]. Further, the inhibitory effect of H_2_S on the absorption of Al^3+^ may be related to an increase in the secretion of citrate. In rice, the expression of *OsFRDL4*, a gene that regulates the efflux of citrate, was significantly up-regulated by NaHS treatments in Al-stressed conditions, and a simultaneous increase in citrate secretion from roots was found in the NaHS-pretreated group compared with the untreated Al-stressed plants [81]. Similarly, research on barley has shown that H_2_S could promote citrate secretion in roots and could up-regulate the expression of the citrate transporter gene (*HvAACT1*) when the plants were subjected to Al stress [80]. The increase in the rate of citrate secretion reduced the deposition of Al on the surface of roots. Therefore, the promotion of citrate secretion by H_2_S could lead to a reduction in the Al content of roots. Moreover, H_2_S-induced antioxidant-related enzyme activity also contributed to the mitigation of aluminum toxicity. The NaHS pretreatment significantly increased SOD, APX, CAT, and POD activity in Al-stressed rice [81]. Similarly, H_2_S was found to enhance SOD, CAT, POD, and GR activity in roots under Al stress in barley [94].

### 3.3. Salinity

According to previous studies, the inhibition of root growth by salinity (NaCl) stress could be attributed (at least in part) to a decrease in K^+^ concentrations and the K^+^/Na^+^ ratio in the cytoplasm. This would have disrupted ion homeostasis and hence caused cell death [95]. It displayed a net K^+^ efflux after exposure to NaCl, whereas NaHS could restrict the NaCl-induced K^+^ efflux in both salt-tolerant or salt-sensitive grape roots. Furthermore, H_2_S promoted Na^+^ efflux and the influx of H^+^ by up-regulating the Na^+^/H^+^ antiport system to maintain the plasma membrane (PM) polarity, thereby reducing the K^+^ loss by inhibiting PM depolarization-activated K^+^ channels [88]. K^+^ and Na^+^ homeostasis was an important adaptation by plants to salt stress. Researchers found that H_2_S significantly reduced the Na^+^ content and Na^+^/ K^+^ ratio in wheat roots. The H_2_S facilitated the exclusion of Na^+^ and absorption of K^+^ by regulating selective absorption and transport of K^+^ over Na^+^ [87]. The content of H_2_S in a *Brassica napus* hybrid was more than that of the two parents. When exposed to salt stress, the expression of *NHX1* (Na^+^/H^+^ antiporter), *AKT1* (inward-rectifying potassium channel), and *HAK5* (potassium transporter) was significantly higher in the hybrid, in which, the Na^+^ content and Na^+^/ K^+^ ratio was reduced, and the K^+^ content increased. The hybrid, therefore, had a higher salt tolerance than the parents. However, these beneficial effects in the hybrid were eliminated by HT and PAG [96]. These results indicated that H_2_S improved the salt tolerance of plants by maintaining Na^+^ and K^+^ homeostasis. Other studies have shown that the regulation of Na^+^ and K^+^ homeostasis in salt-stressed plants by H_2_S involved the Ca^2+^ and NO signal pathways. Ca^2+^ and H_2_S had a synergistic effect on the induction of the Na^+^/H^+^ antiport system in mung bean roots. In contrast, the HT treatment negated the beneficial effects of Ca^2+^ on salt stress. Furthermore, a supplementation of Ca^2+^ could enhance H_2_S biosynthesis through promoting a cysteine pool. This implied the downstream functioning of H_2_S during the Ca^2+^-mediated regulation of plant adaptive responses to NaCl stress [97]. Both NO and H_2_S could increase the K^+^/Na^+^ ratio in alfalfa roots, whereas the treatment with cPTIO reduced the H_2_S-induced K^+^/Na^+^ ratio and antioxidant capacity of H_2_S [85]. When barley roots were exposed to salt stress, H_2_S could decrease the net K^+^ efflux by increasing the transcriptional expression of *HvAKT1* (inward-rectifying potassium channel) and *HvHAK4* (a high-affinity K^+^ uptake system), promote Na^+^ export by increasing the expression of PM H^+^-ATPase (*HvHA1*) and Na^+^/H^+^ antiporter (*HvSOS1*), and transfer excess Na^+^ into vacuoles by increasing the gene expression of vacuolar Na^+^/H^+^ antiporter (*HvVNHX2*), H^+^-ATPase subunit β (*HvVHA-β*), and the accumulating vacuolar Na^+^/H^+^ antiporter (NHE1) protein. However, these effects induced by H_2_S were quenched by the addition of cPTIO [86]. These results mean that the H_2_S is upstream of NO in order to maintain ion homeostasis and improve salt tolerance. However, Da Silva et al. (2018) [89] proposed that H_2_S may act downstream of NO in the mitigation of salt-induced oxidative stress. Researchers found that, after treatment with NaCl, the accumulation of H_2_S in tomato roots occurred later than the accumulation of NO, and that NO could increase the expression of the H_2_S synthesis gene (*L-DES*) and H_2_S production, whereas H_2_S could not induce the accumulation of NO. H_2_S and NO have shown complex interactions when regulating other physiological processes [98]. Therefore, the relationship between H_2_S and NO in plant root growth regulation under salt stress needs more research and discussion.

### 3.4. Hypoxia

Hypoxia leads to root cell death. However, H_2_S could reduce the rate of root tip cell death by inducing antioxidant enzyme activity and by inhibiting ACC oxidase (ACO) activity and ethylene production [90]. H_2_S also promoted endogenous Ca^2+^ accumulation and the Ca^2+^-dependent activity of alcohol dehydrogenase (ADH). It therefore improved the antioxidant defensive capabilities of the plants, and thus increased the rate of maize root tip cell survival in hypoxic conditions [91]. Subsequent studies have shown that the regulation of root tip cell death by H_2_S mediated the NO signal pathway. The NO-induced tolerance of hypoxia was enhanced by the application of NaHS, but was eliminated by HT [91].

## 4. Conclusions and Future Prospects

The effects of H_2_S on plant root growth and development have been widely recognized. In this review, we summarized the regulatory effects of H_2_S on lateral roots, adventitious roots, primary roots, root hairs, and root nodules. The mitigation effect of H_2_S on root growth under abiotic stress was also discussed here. Hydrogen sulfide interacts with a variety of other signals to regulate root growth. These signals mainly included auxin, NO, CO, ROS, and CH_4_. In addition, there are many genes involved in H_2_S-regulated root growth. However, there are still many issues that need to be clarified to explain how H_2_S regulates root growth. For example, H_2_S interacts with other signal molecules to regulate root growth, so finding the key genes that connect H_2_S and other signal molecules is crucial for understanding the complex interactions between H_2_S and signal molecules. Previous studies have found that many genes contribute to the regulation of H_2_S during root growth and development. A great number of genes involved in the regulation of H_2_S on root systems were identified through transcriptome and proteome, but the involvement of these genes was based on the effects of H_2_S on their expression. The importance of these genes to the H_2_S-regulated root growth pathway requires further functional verification. Finally, in recent years, studies have found that H_2_S could directly regulate the S-sulfhydration of proteins by converting Cys-SH to Cys-SSH. This affected the activity of proteins, and, thus, mediated plant growth and development and responses to stresses [99,100,101,102,103]. The ACTIN2 protein, associated with the development of root hair, has been found to be S-sulfhydrated at Cys-287 by H_2_S, thereby mediating H_2_S-regulated root hair growth [29]. This implied that there might be more proteins involved in root development that are S-sulfhydrated by H_2_S that still need to be identified.

## Figures and Tables

**Table 1:** The role of hydrogen sulfide in root during development and its interaction with other signals.

Plant Species	Signal Involved	Root Response	Reference
*Ipomoea batatas*, *Salix matsudana*, *Glycine max*	Auxin and NO	Adventitious root formation	[20]
*Cucumis sativus*	HO-1/CO	Adventitious root formation	[21]
*Cucumis sativus*	Methane	Adventitious root development	[22]
*Solanum lycopersicum*	Auxin	Lateral root formation	[23]
*Capsicum annuum*	Cinnamaldehyde	Lateral root formation	[24]
*Solanum lycopersicum*	H_2_O_2_	Lateral root formation	[25]
*Solanum lycopersicum*	Methane	Lateral root formation	[26]
*Kandelia obovata*	Brassinosteroid, carbohydrate metabolism, cellular redox homeostasis, protein metabolism, secondary metabolism, and amino acid metabolism	Lateral root development	[27]
*Arabidopsis*	ROS, NO, MPK6	Primary root growth	[28]
*Arabidopsis*	Actin dynamics	Root hair growth	[29]
*Glycine max*	Nitrogen-fixation ability	Nodulation	[30]
*Glycine max*	Nitrogen-fixation ability	Nodulation	[31]
*Arabidopsis*	Actin-dependent auxin transport	Root development and growth	[32]
*Prunus persica*	Auxin biosynthesis, transport, and signal transduction.	Root development and growth	[33]
*Fragaria* × *ananassa*	H_2_O_2_ and soluble sugar accumulation	Root development and growth	[34]

**Table 2:** Hydrogen sulfide promotes root growth and its regulation mechanism under abiotic stress.

Abiotic Stress	H_2_S Action	Plant Species	Reference
Cadmium	H_2_S improved oxidation resistance, and NO was involved in the NaHS-induced alleviation of Cd toxicity	*Medicago sativa*	[67]
	H_2_S removed excessive ROS and reduced cell oxidative damage	*Brassica rapa*	[68]
	H_2_S inhibited the ROS burst, and H_2_S-Cys cycle system plays an important role in it	*Arabidopsis*	[69]
	H_2_S mediated the phytotoxicity of Cd by regulating UPB1s-modulated balance between H_2_O_2_ and O_2_^−^	*Brassica rapa*	[70]
	H_2_S relieved-Cd stress was involved in MeJA signal	*Setaria italica*	[71]
	H_2_O_2_ raised H_2_S content in root tissues independently from the desulfhydrase activity, and protected V-ATPase	*Cucumis sativus*	[72]
	H_2_S reduced Cd uptake/translocation and decreased MDA, H_2_O_2_, and O_2_^−^ accumulation	*Hordeum vulgare*	[73]
	H_2_S activated glutathione biosynthetic and AsA-GSH cycle enzymes, and maintained redox status of ascorbate and glutathione	*Solanum lycopersicum*	[74]
	H_2_S inhibited Cd-induced cell death by reducing ROS accumulation, activating the antioxidant system, inhibiting mitochondrial Cyt c release, and reducing the opening of the MPTP	*Cucumis sativus*	[75]
	H_2_S improved Cd tolerance by modulating growth biomarkers and antioxidative system	*Brassica rapa*	[76]
	H_2_S operates downstream of CH_4_, enhancing tolerance against Cd stress	*Medicago sativa*	[77]
Chromium	H_2_S increased Cys accumulation by up-regulating the Cys generation-related genes, enhanced glutathione generation, and activated phytochelatins (PCs) synthesis	*Arabidopsis*	[78]
	H_2_S improved the physiological and biochemical attributes of Cr-stressed plants, and decreased Cr content in different parts of Cr-stressed plants	*Brassica oleracea botrytis*	[79]
Aluminum	H_2_S protected plants against Al toxicity by inducing the activities of antioxidant enzymes, increasing citrate secretion and citrate transporter gene expression, and enhancing the expression of PM H^+^-ATPase.	*Hordeum vulgare*	[80]
	H_2_S alleviated Al toxicity by decreasing the Al content in the apoplast and symplast	*Oryza sativa*	[81]
Lead	H_2_S lowered the Pb concentration in roots, improved the cell structure, and presented the well-developed nucleus with continuous cell membrane	*Brassica napus*	[82]
	H_2_S alleviated Pb toxicity by improvement of nitrate reductase activity and glutathione content and regulation of amino acids metabolism	*Zea mays*	[83]
Nickel	H_2_S induced Ni tolerance that required the entry of extracellular Ca^2+^ into cells across the plasma membrane and the mediation of intracellular CaM	*Cucurbita pepo*	[84]
Salt	H_2_S enhanced plant responses against salinity stress by reducing oxidative damage, which might have a possible interaction with NO	*Medicago sativa*	[85]
	H_2_S increased salt tolerance by maintaining Na^+^ and K^+^ ion homeostasis, which was mediated by NO signal	*Hordeum vulgare*	[86]
	H_2_S alleviated growth inhibition by maintaining a lower Na^+^ concentration under NaCl stress via the regulation of NSCCs and SOS1 pathways	*Triticum aestivuml*	[87]
	H_2_S up-regulated the Na^+^/H^+^ antiport system, which promoted exchange of Na^+^ with H^+^ across the PM and simultaneously restricted the channel-mediated K^+^ loss	*Populus euphratica* and *Populus popularis*	[88]
	H_2_S acts downstream of NO in the mitigation of NaCl-induced oxidative stress	*Solanum lycopersicum*	[89]
Hypoxia	H_2_S protected root tip cell membranes from ROS damage induced by hypoxia, and stimulated a quiescence strategy through inhibiting ethylene production	*Vigna radiata*	[90]
	H_2_S enhanced endogenous Ca^2+^ levels, as well as the Ca^2+^-dependent activity of alcohol dehydrogenase (ADH), improved the capacity for antioxidant defense, and thus increased the NO-induced hypoxia tolerance in maize	*Zea mays*	[91]

## Data Availability

Not applicable.
